# The Compensatory Effect of S375F on S371F Is Vital for Maintaining the Infectivity of SARS‐CoV‐2 Omicron Variants

**DOI:** 10.1002/jmv.70242

**Published:** 2025-03-10

**Authors:** Shuo Liu, Pan Liu, Qiong Lu, Yanru Shen, Li Zhang, Ziteng Liang, Yuanling Yu, Weijin Huang, Youchun Wang

**Affiliations:** ^1^ Changping Laboratory Beijing China; ^2^ Chinese Academy of Medical Sciences and Peking Union Medical College Beijing China; ^3^ CAS Key Laboratory of Infection and Immunity, National Laboratory of Macromolecules, Institute of Biophysics, Chinese Academy of Sciences Beijing China; ^4^ Division of HIV/AIDS and Sexually Transmitted Virus Vaccines Institute for Biological Product Control, National Institutes for Food and Drug Control (NIFDC) Beijing China

**Keywords:** “tug of war” model, compensatory effect, Omicron strain, S371F + S375F, SARS‐CoV‐2, Virus‐like particle (VLP)

## Abstract

The emergence of Omicron variants dramatically changed the transmission rate and infection characteristics compared to previously prevalent strains, primarily due to spike protein mutations. However, the impact of individual mutations remained unclear. Here, we used virus‐like particle (VLP) pseudotyped to investigate the functional contributions by 12 common mutations in the spike protein. We found that the S371F mutation in the receptor binding domain (RBD) of spike protein led to a 5‐ and 10‐fold decrease of ACE2 utilization efficiency and viral infectivity, respectively, accompanied by a 5‐ to 11‐fold reduction of neutralization sensitivity to monoclonal antibodies. However, the S375F mutation in the RBD had a compensatory effect, rescuing the infectivity of the S371F Omicron variant. Based on molecular dynamics simulations, we proposed a “tug of war” model to explain this compensation phenomenon. These results provide a comprehensive and dynamic perspective on the evolution of this important pandemic virus.

## 1 Introduction

The Omicron variant (B.1.1.529) was first detected in South Africa and Botswana, after which it was designated as the fifth variant of concern (VOC) by the World Health Organization (WHO) on November 26, 2021 [1]. The Omicron variant quickly spread worldwide and became the dominant pandemic strain of SARS‐CoV‐2, surpassing previously spreading variants. Over time, the Omicron variant has diversified into multiple sublineages, including BA.1, BA.2, BA.3, BA.5, BF.7, BQ.1.1, XBB.1.5, CH.1.1, EG.5.1, and BA.2.86, all of which are characterized by increased transmissibility, infectivity, and resistance to neutralizing antibodies elicited by prior infection or vaccination [2, 3, 4, 5]. These distinctive viral characteristics are associated with mutations in the Spike (S) protein of the Omicron strain. The S protein is located on the surface of the virion in trimeric form, composed of S1 and S2 subunits, which are highly glycosylated. The S1 subunit contains a crucial but highly variable receptor‐binding domain (RBD), responsible for the binding of the virus to the ACE2 receptor, making it essential for viral invasion and cellular tropism. By contrast, the S2 subunit is relatively conserved and associated with membrane fusion [6, 7]. During the maturation process, S protein undergoes two proteolytic cleavage steps. The first cleavage occurs during the transport of the S protein from the endoplasmic reticulum to the Golgi apparatus after intracellular expression in viral‐producer cells, mediated by the Furin protease. The virions are then able to enter target cells through two distinctive pathways, one of which is mediated by S protein binding to the ACE2 receptor This exposes the S2 subunit, where the second cleavage occurs by recruiting TMPRSS2 protease. By contrast, during the process of viral cell entry through endocytosis, cleavage is performed by host protease cathepsin L. The second cleavage induces fusion of the virion with the host cell membrane [8, 9, 10, 11]. The cleavage of the S protein by TMPRSS2 protease is a key factor that induces the formation of fused multicellular syncytia, which are associated with an increase in disease severity [12, 13].

The receptor binding domain (RBD) of S protein largely determines viral transmissibility and immune escape process. The S375F substitution was found to affect the cleavage of the S protein, resulting in reduced abundance of the S2 truncation product [14, 15, 16]. The Q493R mutation has an epistatic effect, and in combination with the F456L mutation, it contributed to the emergence of the EG.5 variant [17]. The N501Y mutation exhibited an epistatic effect in enhancing the binding affinity of the virus for the receptor, and in combination with other mutations that affect the ACE2‐binding affinity, such as Q498R, resulted in enhanced virus‐cell interactions [18].

Previous studies have identified several S protein mutations in Omicron strains that affect the viral cell entry process. The K417N, N440K, L452R, T478K, E484A, F486V/S/P, and N501Y mutations affect the ACE2 binding affinity and lead to immune escape of the Omicron strain [19, 20].

In this study, we mainly utilized VLP pseudotyped viruses with the complete structural genes of SARS‐CoV‐2, encoding S, E, M, and N proteins [21]. In addition to four structural proteins, it also contains the PS9 plasmid with a reporter gene. This plasmid, which contains a 1092‐bp fragment of nsp15–nsp16, plays a crucial role in the assembly of the VLP pseudotyped viruses, resulting in the highest viral titer. The truncated form of PS9, named PS966, produces a higher titer of VLP pseudotyped virions [22].

Recent studies mainly focused on the impact of single mutations on viral immune escape, tissue‐specificity during infection, as well as the combined impact of ACE2 affinity‐enhancing mutations with other sites. We previously found that several mutations (e.g., S371L, S371F) were associated with decreased infectivity (unpublished data). Consistent with the research findings of Pastorio et al., S371L/F resulted in reduced infectivity of BA.1 and BA.2 strains [23]. However, it is unknown how the Omicron strain compensates for these reduced infectivity sites to maintain high infectivity. Here, we utilized the SARS‐CoV‐2 VLP pseudotyped virus platform to investigate the infection characteristics of Omicron variants, aiming to identify sites that can compensate for the reduced infectivity of S371F and explain the mechanism of this compensation. We found that mutation S371F in the RBD of spike protein led to a 5‐ and 10‐fold decrease of ACE2 utilization efficiency and viral infectivity, respectively. At the same time, the neutralizing activity of monoclonal antibodies targeting epitopes 1 and 4 was attenuated 5‐ to 11‐fold by the S371F mutation. However, the S375F mutation in the RBD had a compensatory effect when combined with S371F, functionally rescuing the corresponding Omicron variants. Notably, the S375F mutation first appeared in the BA.1 variant, and the S371F mutation first appeared in the BA.2 variant, but the S371F and S375F mutations always appeared simultaneously after the latter, and persisted in all subsequent Omicron variants.

## 2 Materials and Methods

### 2.1 Plasmids

Cloning plasmids encoding structural proteins: Plasmids encoding S, N, SARS‐CoV‐2‐M‐IRES‐E, and PS966 were constructed based on the pcDNA3.1 backbone. The fully synthetic codon‐optimized NA sequences of E, M, and N were PCR‐amplified from corresponding plasmids that were provided by General Biotech (Anhui) Co. Ltd, Chuzhou City, China. Site‐directed mutagenesis was utilized to generate variants of S protein.

### 2.2 Cell Lines

Cells were maintained in Dulbecco's modified Eagle's medium (DMEM; HyClone, Logan, UT, USA) in a humidified incubator at 37°C with 5% CO_2_. The 293T (CRL‐3216; American Type Culture Collection [ATCC], Manassas, VA) and 293T‐hACE2 cells were maintained in our laboratory.

### 2.3 Monoclonal Antibodies

Thee 10 monoclonal antibodies were generously donated by Professor Wang Xiangxi from the Institute of Biophysics, Chinese Academy of Sciences.

### 2.4 Packaging and Titration of VLP Pseudotyped Viruses

To produce the SARS‐CoV‐2 VLP pseudotyped viruses, plasmids encoding Cov2‐N, CoV‐M‐IRES‐E, CoV‐2‐Spike, and Luc‐PS966 were combined at specific ratios in a total dose of 13 µg of DNA. The mixture was then diluted in 250 µL of Opti‐MEM before adding P3000 Reagent and Lipofectamine 3000 Transfection Reagent for DNA complexation. After a 20‐min incubation period at 37°C, the transfection mixture was gently added to 293T cells in DMEM supplemented with fetal bovine serum (FBS) and penicillin/streptomycin. The medium was refreshed at 12 h posttransfection, while the VLP pseudotyped virus‐containing supernatant was harvested at 48 h after transfection and filtered through a 0.45 µm pore‐size syringe filter.

### 2.5 Luciferase Assay

In an opaque white 96‐well plate, 50 µL of supernatant containing the SARS‐CoV‐2 VLP pseudotyped virus was combined with 50 µL of a cell suspension in each well. The cells were allowed to adhere and internalize the VLP pseudotyped viruses overnight. The medium was aspirated from the cell culture dish after approximately 18 h. Subsequently, 100 µL of chemiluminescent substrate was added to each well. The reaction was allowed to continue for 2 min before measuring the luminescence signal using a luminometer.

### 2.6 Neutralization of VLP Pseudotyped Virus

Each well of a 96‐well plate was filled with a 100 µL sample, diluted at a ratio of 1:10, and subsequently serially diluted in threefold increments. Following this, 50 μL of VLP pseudotyped virus suspension (2050 TCID50) was combined with the diluted samples in a separate 96‐well plate. The mixture underwent incubation at 37°C for a period of 1 h, before being co‐incubated with 293T‐ACE2‐Furin cells (3 × 10^4^ cells/well) at 37°C in a humidified environment with 5% CO_2_. The chemiluminescence signals were assessed in relative luminescence units (RLU) following 18 h of incubation. The IC50 value was determined using the Reed–Muench method.

### 2.7 Molecular Dynamics Simulations

The initial model of D614G RBD was extracted from structure with PDB ID 7T67. Models of S371F RBD, S375F RBD, and S371F + S375F RBD were obtained by mutating the corresponding residues in Coot. Briefly, we generated the inputs in CHARMM‐GUI for the GROMACS simulation package before final stimulation. After PDB verification, specifying the box size, water model (TIP3P), addition of ions to neutralize the systems, setting periodic boundary conditions and specifying the force field (OPLS‐AA/M), the data generated was submitted to GROMACS‐2022 for energy minimization, NVT equilibration, NPT equilibration and 50 ns MD of simulation. The NVT and NPT ensembles were completed using the Nose‐Hoover method at 300 K and the Parinello‐Rahman algorithm at 1 bar to equilibrate the temperature and pressure, respectively. The final 2 ns frames were extracted to calculate the RMSF.

### 2.8 Statistical Analysis

Data were analyzed using the GraphPad Prism 8.0 software (GraphPad, San Diego, CA). For comparisons between two data sets, we utilized the unpaired two‐tailed Student's *t*‐test. To examine multiple datasets, one‐ or two‐way ANOVA accompanied by Dunnett's multiple comparisons test was applied. The experimental data were based on at least two independent trials. The results are expressed as means ± standard deviations (SD). Significance levels are indicated as follows: *p < 0.05, **p < 0.01, ***p < 0.005, and ****p < 0.001.

## 3 Results

### 3.1 Omicron Variants Showed No Alternation in ACE2 Utilization

SARS‐CoV‐2 VLP pseudotyped virus contains four structural proteins, S, M, E, and N. We previously packaged a high titer of SARS‐CoV‐2 VLP pseudotyped virions by optimizing the expression of the latter three proteins. Here, we successfully constructed 15 SARS‐CoV‐2 VLP pseudotyped viruses with various S protein mutations. To examine the infectivity, two cell lines (293T cells with/without ACE2‐overexpresseion) were infected with the same dose of pseudotyped virions. The efficiency of viral utilization of ACE2 was represented by the ratio of luminescence on corresponding protein‐overexpressing 293T cells.

**Figure 1:**
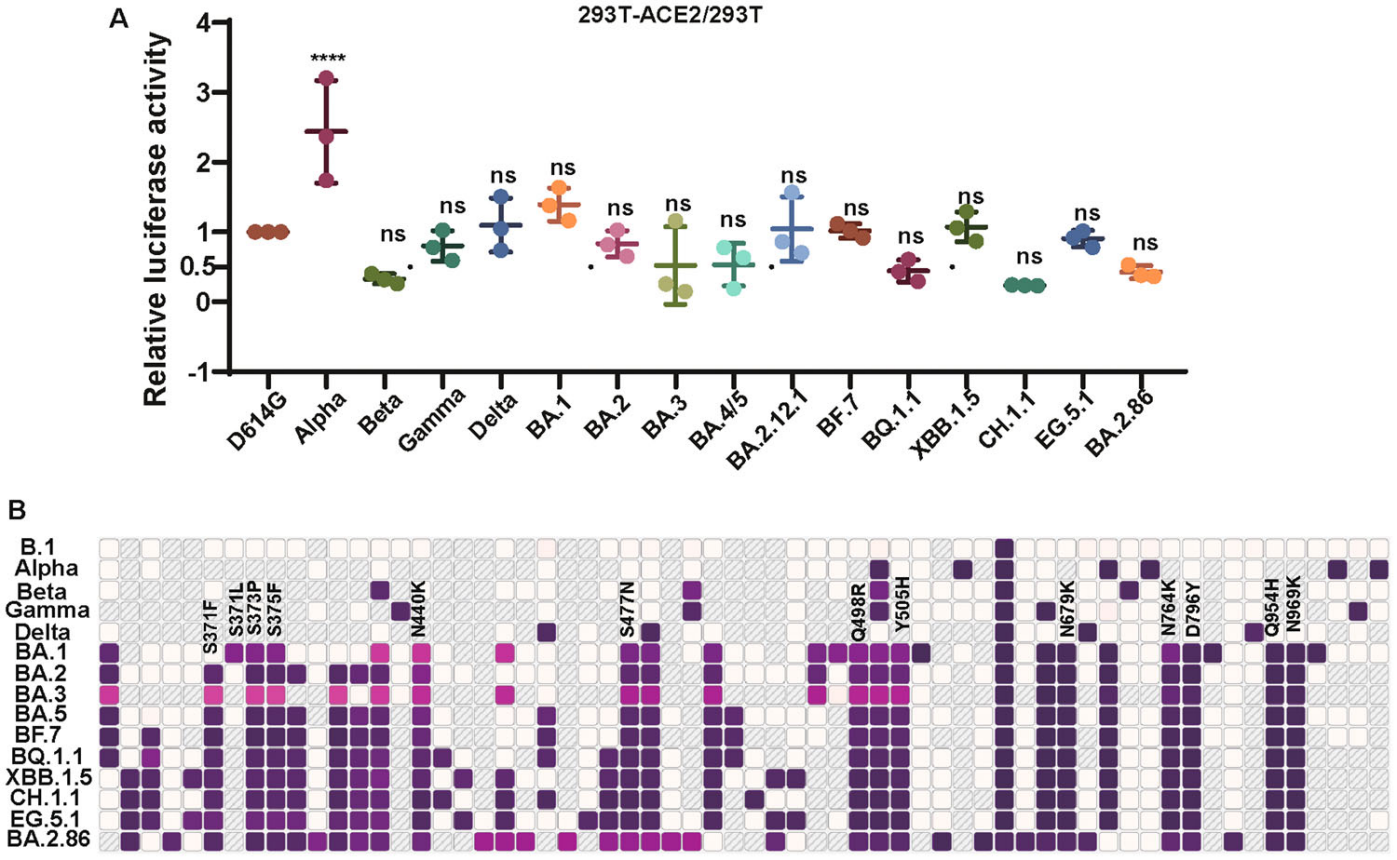
Comparation of early variants of concern (VOC) and the Omicron variant. (A) The ratio of luciferase activity between the SARS‐CoV‐2 variants and the D614G strain. (B) Diagram illustrating mutation sites that are absent from early variants of concern (VOC) but shared by the Omicron variants. To statistically analyze multiple sets of data, one‐ or two‐way ANOVA and Dunnett's multiple comparisons test were utilized. The experimental data were derived from at least two repeated trials. The results are presented as means ± standard deviations (SD). Significance thresholds: *p < 0.05, **p < 0.01, ***p < 0.005, and ****p < 0.001.

We found that the ACE2 utilization efficiency of Omicron series VLP pseudotyped viruses showed a twofold change compared to the D614G virus strain, while the utilization efficiency of the Alpha variant increased by approximately 2.5 times (Figure 1A). Next, to determine the point mutations that affected the ACE2 utilized efficiency of Omicron variants, we identified 12 mutation sites on the S protein of these strains, which were absent in earlier VOCs but commonly expressed in the Omicron lineage (Figure 1B).

### 3.2 S371F Led to a Fivefold Reduction of ACE2 Utilization, While the Combination of S375F With S371F Increased the Utilization Efficiency of ACE2, Instead of N969K

**Figure 2:**
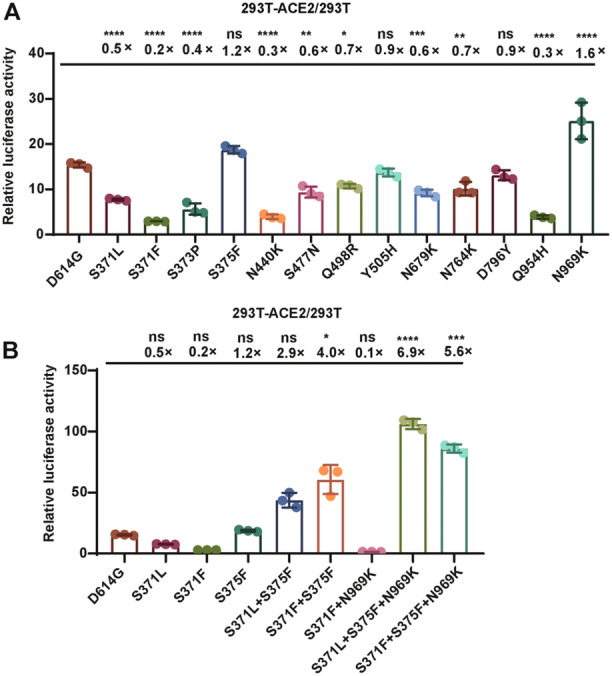
Comparison between the Omicron variant with combined mutations and the D614G strain. (A) The ratio of luciferase activity between the Omicron variant with shared mutation sites and the D614G strain. (B) The ratio of luciferase activity between the Omicron variant with combined mutation sites and the D614G strain. To statistically analyze multiple sets of data, one‐ or two‐way ANOVA tests and Dunnett's multiple comparisons test were utilized. The experimental data are derived from at least two repeated trials. The results are presented as means ± standard deviations (SD). Significance thresholds: *p < 0.05, **p < 0.01, ***p < 0.005, and ****p < 0.001.

We constructed 13 SARS‐CoV‐2 VLP pseudotyped viruses based on the D614G strain, whereby the S371L, S371F, S375F, N440K, S477N, Q498R, Y505H, N679K, N764K, D796Y, Q954H, and N969K mutations were individually introduced to test whether they affect the usage of ACE2. Among the 13 mutated sites, only the S proteins that carried S375F and N969K mutations showed increased ACE2 utilization, whereby the efficiency was increased 1.2‐ and 1.6‐fold, respectively. The ACE2 utilization efficiency was reduced when other single site mutations occurred, whereby S371F led to the most significant decline of up to fivefold (Figure 2A).

Previous data showed that the VLP pseudotyped viruses constructed based on Omicron variants exhibit slight variation of ACE2 utilization efficiency compared to the D614G strain (Figure 1A). We found that of the 13 single mutation of S protein, only S375F and N969K were capable of enhancing ACE2 utilization. Based on this, we hypothesized that S375F and N969K have a certain rescuing effect on the reduced utilization efficiency of S371F. To solve this, we constructed the S371F + S375F, S371F + N969K, and S371F + S375F + N969K combinations, which revealed that the simultaneous occurrence of S375F and S371F resulted in a fourfold increase of the ACE2 utilization efficiency, even surpassing the ACE2 utilization efficiency when only S375F was presented, by 20% (Figure 2B). By contrast, S371F + N969K led to a 10‐fold reduction of ACE2 utilization efficiency. Furthermore, after combining S371F + S375F with N969K, the ACE2 utilization efficiency increased 5.6‐fold (Figure 2B).

### 3.3 S375F Rescued the Reduced Infectivity Caused by S371F

**Figure 3:**
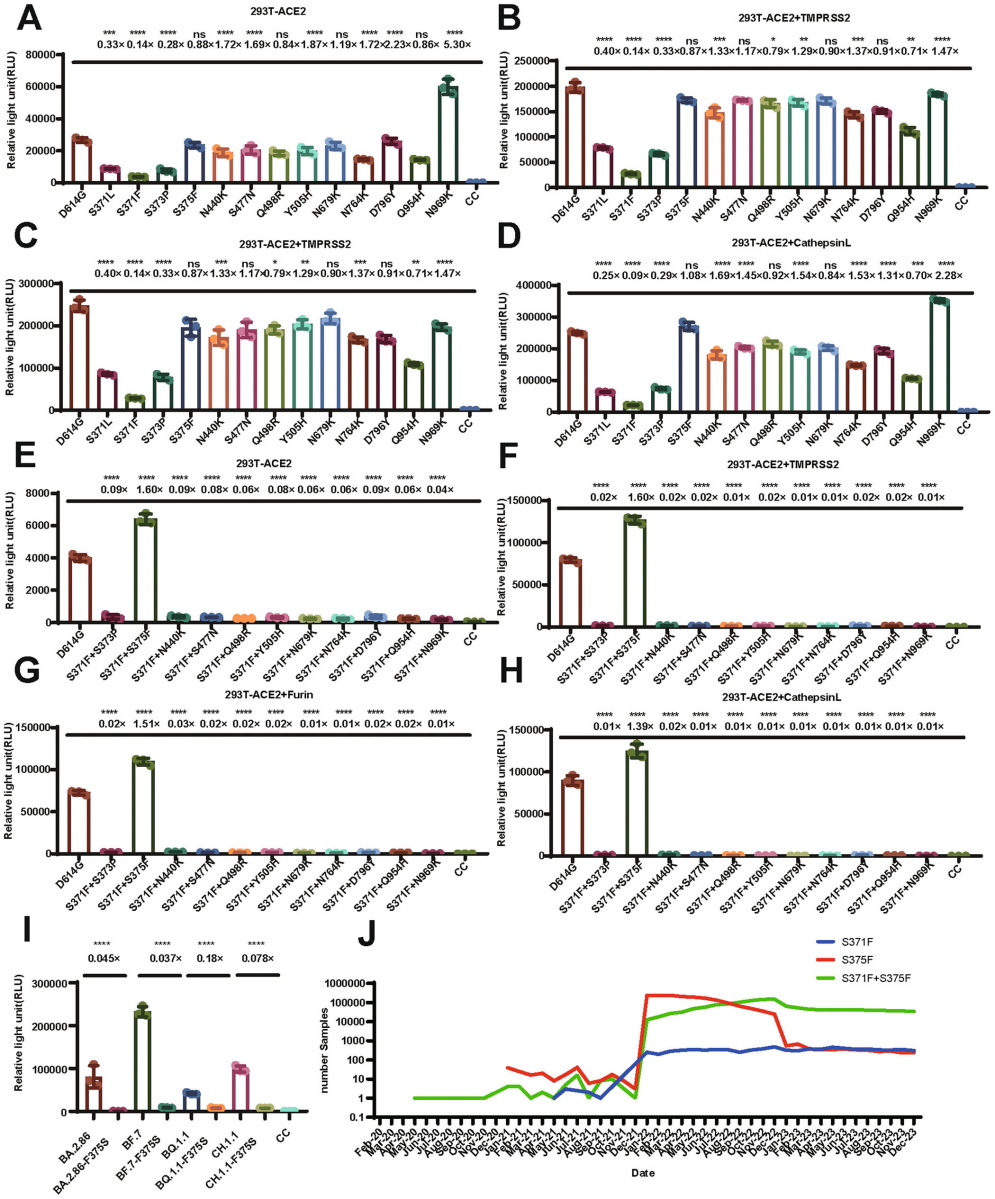
Comparison of the infectivity of Omicron variants with shared single point mutations and combined mutations. The D614G strain was used as a negative control. (A–D) Ratio of infectivity of the Omicron variants with shared single point mutations to the D614G strain in four different cell lines. (E–H) Impact of combining S371F with other shared Omicron mutation sites on infectivity. (I) Comparison of infectivity between the BA.2.86, BF.7, BQ.1.1, and CH.1.1 variants with the F375S mutation. (J) Changes in the abundance of viral sequences containing only S371F, S375F, or both S371F and S375F mutations. To statistically analyze multiple sets of data, one‐ or two‐way ANOVA and Dunnett's multiple comparisons test were utilized. The experimental data are derived from three repeated trials. The results are presented as means ± standard deviations (SD). Significance thresholds: *p < 0.05, **p < 0.01, ***p < 0.005, and ****p < 0.001.

Because viral cell invasion capacity is impacted not only by the utilization of ACE2 but also the infectivity, we performed a luciferase assay to evaluate the infectivity of the VLP pseudotyped viruses. Accordingly, we analyzed the infectivity of the 13 mutant strains and demonstrated that both the S371L and S373P mutations resulted in a 3.3‐fold attenuation of infectivity, while S371F led to a 10‐fold decrease relative to the D614G strain in 293T cells overexpressing ACE2 (Figure 3A). Conversely, the infectivity of the N969K mutant VLP pseudotyped virus was enhanced 5.3‐fold (Figure 3A). Akin to the infection assay in ACE2 single overexpression cells, the infectivity of VLP pseudotyped virus was respectively reduced 2.5‐, 10‐, and 3.3‐fold by the S371L, S371F, and S373P mutations when infecting ACE2 + TMPRSS2 overexpressing cells (Figure 3B). Moreover, similar reductions of infectivity were also observed in VLP pseudotyped viruses infecting furin or cathepsin overexpressing cells (Figure 3C,D). To examine whether combined mutations affected the infectivity, we combined S371F with other S protein mutations. The virus titer only regained the level comparable to the D614G strain when S371F was combined with S375F, while other mutant combination resulted in a further depletion of viral infectivity by more than 10‐fold (Figure 3E–H) indicated that S375F has a specific rescuing role for the loss of infectivity observed in S371F. To further verify this conjecture, we conducted reverse mutagenesis to create the S375 mutations in four strains selected from the Omicron lineage, including BA.2.86, BF.7, BQ.1.1, and CH.1.1. The titers of the VLP pseudotyped viruses for these four strains respectively decreased 22.4‐, 26.8‐, 5.6‐, and 12.9‐fold (Figure 3I), demonstrating that S375F is an important mutation for the survival and transmission of Omicron variants. Additionally, we analyzed the relative abundance of the S371F, S375F, and S371F + S375F mutations from 2020 to 2022 and found that, initially, the circulating strains rarely carried these mutations. As the S375F mutation became more prevalent, the abundance of the S371F + S375F combined mutation subsequently also rose, followed by a decrease in the abundance of single‐point S375F sequences, which ultimately appeared only in combined mutations (Figure 3J).

### 3.4 S375F Partially Restored the Immune Escape Capacity of S371F

**Figure 4:**
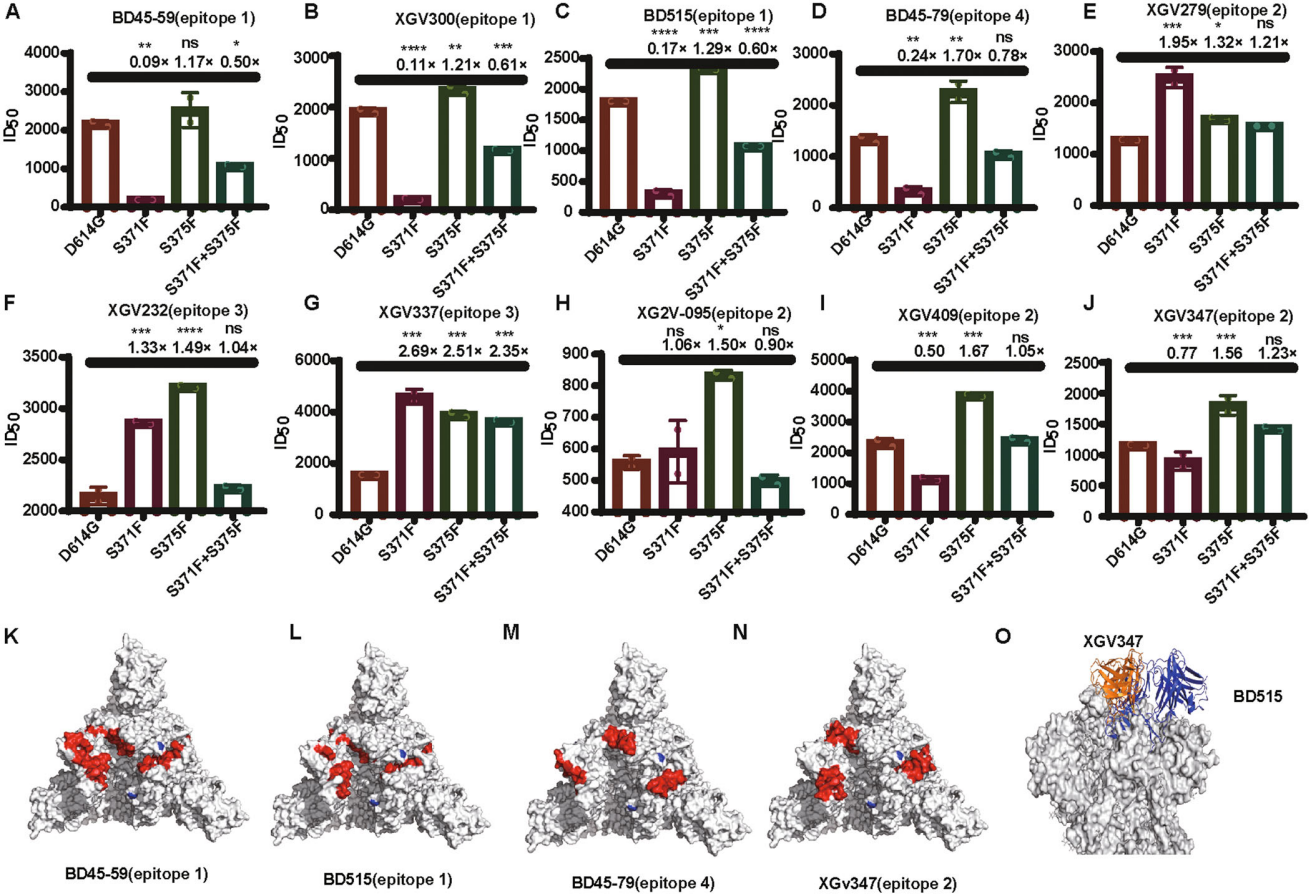
Comparison of the neutralizing activity of 10 monoclonal antibodies against the D614G, S371F, S375F, and S371F + S375F variant strains. (A–D) Four monoclonal antibodies showing decreased neutralizing activity against the S371F mutant strain. (E–J) Six monoclonal antibodies showing no significant decrease of neutralizing activity against the S371F mutant strain. (K–N) Hotspot map of monoclonal antibody epitopes on the spike protein (top view). (O) Side view of the interaction interface between the monoclonal antibody with the spike protein. To statistically analyze multiple sets of data, one‐ or two‐way ANOVA and Dunnett's multiple comparisons test were utilized. The experimental data are derived from at least two repeated trials. The results are presented as means ± standard deviations (SD). Significance thresholds: *p < 0.05, **p < 0.01, ***p < 0.005, and ****p < 0.001.

We selected 10 monoclonal antibodies to evaluate their neutralizing activity against VLP pseudotyped viruses carrying the D614G, S371F, S375F, and S371F + S375F mutations. We found that four of ten monoclonal antibodies exhibited a 5.0‐ to 11.1‐fold reduction of neutralizing activity against the S371F mutant strains (Figure 4A–D), but none of them completely lost neutralizing activity. Other antibodies showed no significant changes of neutralizing activity (Figure 4E–J). Furthermore, three of four attenuated antibodies targeted epitope 1, while the remaining one targeting epitope 4. Among the six antibodies that did not show a significant decrease in neutralizing activity, four targeted epitope 2, while the remaining two targeted epitope 3. We speculated that this might be due to the locations of the epitopes on S protein, as antibodies targeting epitopes 1 and 4 are specific for the closed conformation of the S protein, while epitopes 2 and 3 are located on the protein surface [24]. To prove our hypothesis, we analyzed antibody target hotspots on S protein, with most binding hotspots for epitopes 1 and 4 located inside the spike protein, whereas hotspots for epitope 2 were located on the surface of the S protein (Figure 4K–N). Epitope 1 of monoclonal antibody BD515 is characterized by more side chains inserting into the interior of the spike protein, whereas epitope 2 of monoclonal antibody XGv347 is located on the surface of the spike protein (Figure 4O).

The combination of S375F and S371F caused a 1.3‐ to twofold decrease of neutralizing activity for the 4 tested antibodies (Figure 4A–D). However, compared to the S371F mutation alone, the neutralizing activity was subsequently restored, indicating partial restoration of antigen conformation. Although the combination of S371F and S375F resulted in a reduction of immune escape ability, the combined mutations restored the infectivity of VLP pseudotyped viruses, which increased 1.4‐ to 1.6‐fold (Figure 3E–H). Furthermore, the combined mutations enhanced the affinity of S371F + S375F VLP pseudotyped virus binding to ACE2 fourfold (Figure 2B).

### 3.5 Structural Analysis Revealed the Mechanisms of S375F Rescuing the Infectivity Reduction of S371F and Conformational Change

**Figure 5:**
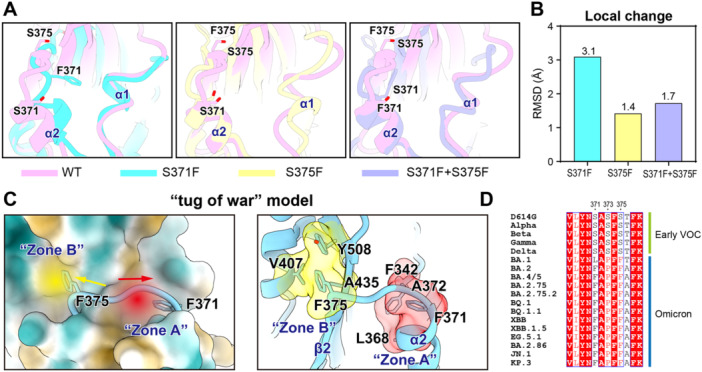
Molecular dynamics simulation of individual S371F and S375F mutations as well as their combinations. (A) Molecular dynamics simulations of the 3 mutations in α1 and α2. (B) RMSD values of the three mutations in α1 and α2. (C) Schematic diagram explaining the proposed “tug of war” mechanism. (D) The mutation status of these three loci in the variant strain.

Residues 371 and 375 are located in the secondary structures alpha1 and alpha2 of RBD. It has been reported that mutations in this region affect the local conformation and even the stability of RBD [25, 26]. To explore the potential structural effects of the S371F and S375F mutations, we performed 50 ns molecular dynamics simulations on D614G RBD and three RBD models harboring the mutation S371F, S375F, and S371F + S375F. Compared with the D614G RBD, the RBD with the S371F mutation presented a highly significant conformational change in the α1 and α2 secondary structures, followed by S371F + S375F. By contrast, S375F only led to a subtle change of the RBD structure (Figure 5A). Furthermore, we quantified the structural variations of α1 and α2 on the D614G RBD using the RMSD (root mean square deviation) metric. The S375F mutant demonstrated the most stabilized structure, with an RMSD value of 1.4 Å. Proteins carrying the combined S371F and S375F mutations showed a decreased RMSD value (1.7 Å) compared to the S371F single mutation (3.1 Å) (Figure 5B), suggesting that S375F might remedy the S371F‐induced local conformational change via an unknown mechanism. To explain this rescuing effect, we proposed a “tug of war” model, whereby F371 is located in the hydrophobic zone A of the RBD that also comprises residues L368, A372 and F342, while F375 is located in hydrophobic zone B, together with residues V407, A435 and Y508 (Figure 5C). The substitution of serine 371 with phenylalanine expanded hydrophobic zone A, thereby inducing a local structural rearrangement of α2 that then approached α1. This was further transmitted to the downstream residues, consequently destabilizing the structure of residue 375 and β2. However, the mutation of residue S375 compensated for this destabilizing effect. Similarly, F375 also expanded hydrophobic zone B, while stabilizing the local conformation of β2 via hydrophobic stacking. In fact, residues 371 and 375 remained conserved in BA.2 and subsequent variants. Moreover, we also found that the T376A mutation further expanded hydrophobic zone B to enhance local stability (Figure 5D).

## 4 Discussion

Since Omicron became the dominant circulating strain worldwide, the transmissibility of SARS‐CoV‐2 was enhanced, which was associated with point mutations of S protein. Previous studies have investigated numerous S protein mutations with positive effects on viral transmission and immune escape, including N501Y, D614G, and P681H [27, 28]. However, several S protein mutations also result in dampened viral infectivity [15], and the mechanism through which SARS‐CoV‐2 compensates for these mutations is still unclear. Based on this, we systematically investigated the utilization efficiency of ACE2 in pseudotyped viruses based on the Omicron variant and previous VOCs. The utilization of ACE2 by Omicron series variants remained efficient, while the point mutation S371F decreased the efficiency by fivefold. Among the 12 point mutations of Omicron variants we evaluated, only S375F and N969K were positively correlated with the utilization efficiency of ACE2 receptor. After combining the N969K and S371F mutations, the infectivity did not recover, but it has been reported that N969K can affect the structure of viral HR1, thus in turn affecting the membrane fusion process [29]. We also combined S371F with S375F, which demonstrated that the efficiency attenuation caused by S371F was only compensated in the presence of S375F. In addition, the combination further enhanced the utilization efficiency of ACE2. L452R and N501Y were also suggested to enhance or compensate the fusogenic or receptor binding activity of S‐protein [18, 30, 31, 32]. Unlike S375F, the combination of N501Y with any mutations was capable of enhancing the binding affinity of S protein for ACE2, while S375F only specifically compensated the decreased infectivity caused by S371F. In addition, we found that the joint mutation of S371L and S375F increased the infectivity 2.9 times, while the double mutation of S371F and S375F increased the infectivity 4.0 times, which may explain the presence of the latter in all mutant strains after BA.2. BA.1 containing S371L and S375F was eliminated, and the subsequent BA.2.86 is also a descendant of BA.2 [23, 33].

Mutation S375F, which was first identified in this study, was able to rescue the attenuated infectivity caused by S371F. We also observed that the combined mutation of S371F and S375F enhanced the ACE2 utilization efficiency, while the single mutation S375F had no significant effects on ACE2 binding. Henceforth, the S371F + S375F combined mutation is becoming dominant in the evolution of Omicron variants, rather than generating S371F or S375F single point mutations. However, unlike the epistatic effects of N501Y, or the global impacts on all mutation sites when combined with D614G, the compensatory effect observed in this study is only present specifically between S375F and S371F. Hence, the rescue effect of S375F might also lead to further negative feedback on S375F, as the substitution of phenylalanine with serine on position 375 decreases the S protein expression level [34]. Moreover, it resulted in inefficient spike cleavage and attenuated fusogenicity of SARS‐CoV‐2, which is dependent on a pi–pi interaction between F375 and H505 of another interprotomer [14]. These results illustrated an intrinsic balance of regulation by S375F. Accordingly, the S375F mutation had a positive effect on viral infection and transmission but negatively impacted S protein production and S2 protein cleavage. Furthermore, the combination of S375F, S371F, and N969K also resulted in an enhancement of ACE2 utilization efficiency, whereas none of the other mutations we introduced in this study had a similar effect. Instead, these mutations counteracted the effects of S375F and N969K, leading to a reduction of ACE2 binding affinity. These results indicate that S375F may also play a balancing role in regulating viral transmission and pathogenicity.

It was reported that the efficacy of the majority of previous antibodies is substantially compromised by three mutational hotspots in the bottom part of RBD (S371L/F, S373P, and S375F), which are found in Omicron BA.1, BA.2, and BA.4/5 [35]. C > U mutations at S371F, S373L, and S375F conferred significant immune escape capacity [36]. However, we found that The S371F mutation led to a decrease of neutralizing titers in monoclonal antibodies targeting epitopes 1 and 4, which could be rescued by the combination of S371F + S375F. This indicates that the S375F mutation has a structural compensatory effect on S371F, repairing the protein malfunction caused by S371F on epitopes 1 and 4 to a certain extent. Viral antibody escape generally occurs through two mechanisms. The first mechanism is based on the mutation of specific amino acids, resulting in protein surface alternations that weaken or abolish antibody binding. This, in turn, leads to complete loss of neutralizing activity in monoclonal antibodies. The second mechanism involves mutations that alter the entire antigen structure, leading to attenuated antibody binding [37]. Unlike the first mechanism, the latter is unable to completely eliminate the neutralizing activity. Accordingly, the S371F mutation might induce antibody escape via the second mechanism [37], by distorting the antibody conformation and reducing the neutralizing activity. Cryo‐EM structural analysis and MD simulations by Wieczo et al. revealed that residues 371, 373, and 375 favor tighter packing and support the neighboring RBD [16, 35], which is parallel to the conformation and antibody escape studies of Zhao et al., who found that the S371‐S373‐S375 loop mediates antibody escape by reducing the accessibility of epitopes to targeting antibodies [36, 38]. Using molecular dynamics simulations of the S protein trimer, we observed that S371F induced significant conformational changes near the alpha1 and alpha2 helices of the RBD, while the combined mutation S371F and S375F led to a relatively smaller change. This indicates that the additional S375F mutation compensated for the destabilizing effect of S371F, which might be the structural basis for the rescue of infectivity reduction as well as the conformational stabilization of epitopes 1 and 4. Furthermore, we proposed a “tug of war” model to explain this mechanism. In this model, F375 extends the volume of hydrophobic zone B, thereby stabilizing the local conformation of β2 through hydrophobic stacking. We not only observed the S371F‐dependent drastic immune escape as mentioned above, but also discovered and clarified the mechanisms of the escape and rescue by S375F for the first time.

Although S375F leads to reduced S protein expression and S2 protein cleavage, it results in immune escape at the expense of reduced infectivity. However, the combined mutation of S371F + S375F rescued the infectivity of Omicron. The combination of S375F with S371F and N969K also resulted in the virus regaining efficiency in utilizing ACE2. These results contribute to a comprehensive and dynamic perspective on the evolution of the virus.

## 5 Limitations of the Study

All results of this study are based on VLP pseudotyped viruses, and validation is needed using live rescue mutant strains. Further validation of the rescuing effect of S375F on the reduced infectivity caused by S371F using molecular dynamics requires additional structural analysis of the mutants. In this study, we were attempting to perform the protein expression assay, but were unable to obtain the mutants containing S375F.

## Author Contributions

Youchun Wang and Weijin Huang revised the manuscript. Shuo Liu, Pan Liu, Qiong Lu, and Yanru Shen wrote the manuscript and analyzed the experimental data, Shuo Liu, Pan Liu, Qiong Lu, Li Zhang, Ziteng Liang, and Yuanling Yu performed the experiments. All authors have read and approved the final manuscript.

## Conflicts of Interest

The authors declare no conflicts of interest.

## Supporting information

Supporting information.

## Data Availability

All data needed to evaluate the conclusions of the article are present in the article and/or the supplementary materials. The datasets used and/or analyzed in the current study are available from the corresponding author upon reasonable request.
