# Supplementary material for: The Compensatory Effect of S375F on S371F Is Vital for Maintaining the Infectivity of SARS‐CoV‐2 Omicron Variants
Source: J Med Virol. 2025 Mar 10;97(3):e70242. doi: 10.1002/jmv.70242 (PMC11891949; doi:10.1002/jmv.70242)
Supplement: Supplementary file 1 — Supporting information. [file JMV-97-e70242-s001.docx]

**The compensatory effect of S375F on S371F is vital for maintaining the infectivity of SARS-CoV-2 Omicron variants**

*Shuo Liu^1,3,5^, Pan Liu^4,5^ , Qiong Lu^2,5^, Yanru Shen^2,5^, Li Zhang^2^ , Ziteng Liang^2,3^, Yuanling Yu^1^, Weijin Huang^2,*^, and Youchun Wang^1,3*^*

*^1^Changping Laboratory, Beijing;*

*^2^Division of HIV/AIDS and Sexually Transmitted Virus Vaccines, Institute for Biological Product Control, National Institutes for Food and Drug Control (NIFDC), Beijing, China;*

*^3^Chinese Academy of Medical Sciences & Peking Union Medical College, Beijing, China;*

*^4^CAS Key Laboratory of Infection and Immunity, National Laboratory of Macromolecules, Institute of Biophysics, Chinese Academy of Sciences, Beijing, China;*

*^5^These authors contributed equally*

**Correspondence: huangweijin@nifdc.org.cn (W.H.), wangyc@nifdc.org.cn (Y.W.)*


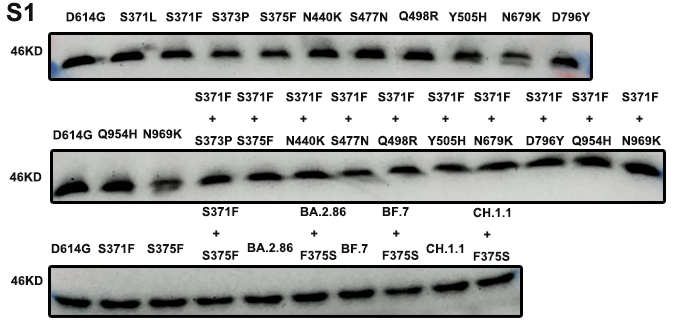


**Fig S1** **Expression of SARS-CoV-2 VLP pseudovirus N proteins with various mutants.** Western blot was performed using antibody specific to SARS-CoV-2 N proteins. Expression of N proteins (46KD) harbor different single and combine mutation sites were shown.

**Materials and methods**

SDS-PAGE and western blotting analysis

Harvested VLP viruses were washed using 5x protein loading buffer (Coolaber, Cat No.: SL1170) then water heating at 95℃ for 5-10 minutes. Subsequently, SDS-PAGE was performed with a 5% stacking gel at 80 V for 20 minutes and an 8%–10% separation gel at 120 V for 1.5 hours. The electrophoresed proteins were then transferred to PVDF membrane at 250 mA for 2 hours. The expression of N protein variants was examined after incubating the PVDF membrane with 1:1000 diluted mouse SARS-CoV-2 (2019-nCoV) Nucleocapsid Antibody, Mouse MAb (Sino Biological, Cat No.: 40588-MM123) as the primary antibody; and developed with goat anti-mouse IgG HRP (Abbkine, Cat.: A21010) at 1:2000 dilution. Intensity of the target protein was quantified by EasySee Western blot Kit (TransGen Biotech, Cat No.: DW101).
